# An in-depth psychometric analysis of the Connor-Davidson Resilience Scale: calibration with Rasch-Andrich model

**DOI:** 10.1186/s12955-015-0345-y

**Published:** 2015-09-23

**Authors:** Víctor B. Arias González, María Teresa Crespo Sierra, Benito Arias Martínez, Agustín Martínez-Molina, Fernando P. Ponce

**Affiliations:** Facultad de Psicología [School of Psychology], Universidad de Talca [University of Talca], 2 norte 685, Av. Lircay s/n, Talca, Chile; Facultad de Educación y Trabajo Social [School of Education and Social Work], Universidad de Valladolid [University of Valladolid], Av. De Belén s/n, Valladolid, España

## Abstract

**Background:**

The Connor-Davidson Resilience Scale (CD-RISC) is inarguably one of the best-known instruments in the field of resilience assessment. However, the criteria for the psychometric quality of the instrument were based only on classical test theory.

**Method:**

The aim of this paper has focused on the calibration of the CD-RISC with a nonclinical sample of 444 adults using the Rasch-Andrich Rating Scale Model, in order to clarify its structure and analyze its psychometric properties at the level of item.

**Results:**

Two items showed misfit to the model and were eliminated. The remaining 22 items form basically a unidimensional scale. The CD-RISC has good psychometric properties. The fit of both the items and the persons to the Rasch model was good, and the response categories were functioning properly. Two of the items showed differential item functioning.

**Conclusions:**

The CD-RISC has an obvious ceiling effect, which suggests to include more difficult items in future versions of the scale.

## Background

Possibly in reaction to models of psychopathology and illness, research on resilience has been gradually increasing over the last 20 years with respect to theory, assessment and implementation [1–3]. Currently, there is little doubt about its potential in healthcare, personal well-being and individual quality of life over the life cycle [4, 5].

The assessment of interventions and programs designed to promote and enhance resilience certainly requires measures with adequate evidence of validity and reliability. Otherwise, it would be impossible to determine the success of a program or to determine who is resilient and to what extent (hence the large differences found in prevalence studies of rates of resilience acrosslevels of risk, revealed -among others- by Haskett et al. [3] and Vanderbilt-Adriance and Shaw [6]). The development of instruments to assess resilience depends on the complexity of the construct and, therefore, on the difficulty of achieving consensus on an operational definition that enjoys sufficient evidence of validity. Although the resilience literature has become remarkably extensive [7], many points of uncertainty still persist and need to be resolved, especially those related to its definition and measurement.

Based on an extensive review of the literature, Windle [8, 9] defined resilience as the process of negotiation, management and adaptation to significant sources of stress or trauma. Protective factors and resources within the individual, as well as the individual’s life and environment, facilitate this ability to adapt to and emerge from adversity. On the other hand, the experience of resilience may be subject to changes over the life cycle. In recent years, several scales and questionnaires have been developed to assess resilience in children, adolescents and adults. The best known instruments that are applied in practice are the *Resilience Scale-RS* [10], the *ER 89* [11], the *Ego Resiliency* scale [12], the *Resilience Attitudes and Skills Profile* [13], the *Connor-Davidson Resilience Scale* (*CD-RISC*) [14], the *Adolescent Resilience Scale* [15], the *Resilience Scale for Adults* [16, 17], the *Dispositional Resilience Scale* [18], the *10-item Connor-Davidson Resilience Scale* [19], the *Youth Resiliency: Assessing Developmental Strengths* (*YR: ADS*) scale [20], the *Resilience Scale of the California Healthy Kids Survey* [21], the *Brief Resilience Scale* [22], the *Child and Youth Resilience Measure* (*CYRM*) [23] and the *Psychological Resilience* scale [24]. All of these scales use a self-report format, and most consist of several factors (with only three of the above scales being unidimensional). Some have been developed for use in clinical settings, whereas others aim to assess resilience in different relational, community and cultural contexts. Windle et al. [7] conducted a review of the psychometric properties of the above scales, assessing their content validity, internal consistency, criteria validity, construct validity, reproducibility (i.e., absolute and relative error measures), responsiveness, presence of floor or ceiling effects and interpretability. The authors awarded a score of 2, 1 or 0 points to each criterion according to whether it was perfectly fulfilled, doubtful or not met. Thus, they prepared a *ranking*, obtaining the *Resilience Scale for Adults* [16, 17] and the *CD-RISC* [14] highest scores. However, the authors indicated that none of the scales exceeded a moderate level of psychometric quality based on a study of the original papers.

Despite the importance of the study of Windle et al [7], we note that the criteria for the psychometric quality of the instruments were based on classical test theory; on the other hand, the source of these criteria does follow some logic, insofar as all of the instruments were developed under its postulates. In our opinion, the authors’ reliance on such determinants to assess the psychometric quality of the instruments—which is represented by the use of Cronbach’s alpha to assess the internal consistency and even the complete reliability of resilience tests—is a limitation of the study. Historically, Cronbach’s alpha is the most commonly used statistic to assess reliability in the literature on psychological research, but it has been seriously challenged in recent years based on the argument that it is not related to the internal structure of the test, given the covariance matrix of items and the typical assumptions about measurement error. It cannot be said, therefore, that Cronbach’s alpha truly measures test reliability (more than internal consistency) or unidimensionality (vid. e.g., Sijtsma [25], Sočan [26] or Ten Berge and Sočan [27]); more powerful alternatives, such as the *Greatest Lower Bound* (*glb*), have been proposed. Another alternative is to calculate the *composite reliability* from the loadings and measurement errors derived from a confirmatory factor analysis of the data, the *ordinal alpha* coefficient (if a factor analysis model is assumed), the *ordinal theta* coefficient (if a principal component analysis model is assumed), or *stratified alpha* from the polychoric correlations to correct the underestimation bias of the coefficient when correlations between variables are high.

The purpose of this paper is to calibrate the *CD-RISC* [14] using the Rasch-Andrich rating scale model, which will be described below. The CD-RISC is inarguably one of the best-known instruments in the field of resilience assessment. It consists of 25 items with five response categories (0 to 4) grouped into five factors. The first factor (8 items) reflects the notion of personal competence, high standards and tenacity. The second factor (7 items) has to do with trust in one’s intuition, tolerance of negative affect, and the strengthening effects of stress. The third factor (5 items) reflects positive acceptance of change and secure relationships. The fourth factor (3 items) reflects control. The fifth factor (2 items) reflects spiritual influences. The scale was validated using different samples (five in clinical settings and one in the community). The authors reported that the scale has high internal consistency, good test-retest reliability and adequate convergent and discriminant validity. However, the CD-RISC has some weaknesses to be described in the following paragraphs.

First, the original factor structure could not be replicated by exploratory or confirmatory factor analysis [19, 28–34]. Second, the existence of the fourth and fifth factors, with three and two items, respectively, is highly questionable. Third, the decision of the authors of the scale [14] of using the Kaiser-Guttmann criterion for deciding the number of factors to retain is questionable because this approach often leads to over-factorization [35]. Moreover, the authors showed a preference for an orthogonal rotation method to analyze scale structure when it would be reasonable to contemplate the possibility that hypothetical dimensions of resilience were correlated, as often occurs with other latent constructs. Fourth, the names of the first three factors are confusing because they include dissimilar concepts. Finally, the multidimensional structure of the scale also seems questionable: Campbell-Sills and Stein [19], after removing items with low or inconsistent loadings or those with overlapping contents concluded that the structure of the scale is unidimensional, retaining only 10 items of the original 25. Subsequent studies have found similar results (e.g., Notario-Pacheco et al. [36], Burns and Anstey [32] and Gucciardi et al. [31]). In the case of the Burns and Anstey study, the authors completed a one-factor solution of 22 items, with adequate fit indices, comparable to those of the reduced 10-item version by Campbell-Sills and Stein [19].

In view of the ambiguities and inconsistencies mentioned and the evidence supporting the unidimensionality of the scale, the purpose of this study focuses on calibrating the CD-RISC using the Rasch Rating Scale Model (RSM), assuming that its structure is unidimensional. Besides being suitable for the study of construct dimensionality, the methods framed in Item Response Theory have obvious advantages over CTT with regard to analysing the psychometric properties of a psychological measurement. These advantages have been widely discussed [37–44]). The most relevant of these findings includes the following: (a) psychometric information does not depend on the sample used; (b) the effectiveness of the scale can be evaluated at each level of the trait or latent variable; (c) it is possible to estimate the precision with which each test (and each individual item) measures different levels of ability/latent traits of the examined participants; and (d) the standard error uses different values throughout the continuum of the latent variable.

To the best of our knowledge, no study has evaluated the psychometric properties of the CD-RISC using IRT despite its advantages. Having precise measures is of utmost importance for numerous reasons, among which we highlight the following. First, results from studies testing structural models are as reliable and valid as those from models explaining how latent variables are measured and, by extension, as reliable and valid as the evaluation instruments used. Second, from the perspective of the clinical application of these evaluations, IRT models provide a considerably deeper knowledge of items that ideally provide precise and valid instruments for the diagnosis, classification, and evaluation of intervention effectiveness.

## Methods

### Participants

A convenience sample of 444 adults (24.5 % men and 75.5 % women) with a mean age of 36.18 years (*SD* =19.5) was used. The mean age of the women was 36.08 years (*SD* =19.2); of the men, 36.58 years (*SD* =20.40). The high dispersion of the sample was due to its being a bimodal distribution (viz., 279 participants were college students, with the remainder (*N* =165) being older participants who had been educated at the University of Experience Program). The analysis of the gender and age variables did not yield significant differences from the expected frequencies (t _(442)_ =0.248, p =0.804), so we accept the equiprobability hypothesis -i.e., independence between the two variables. The participants in this study completed the scale after sign the corresponding consent, adjusted to the Code of Practice for Research at the University of Valladolid (Spain, last update of the Governing Council of 31 January 2013).

### Measures

The Spanish version of the Connor-Davidson Resilience Scale (CD-RISC) was used [14, 45]. The properties and theoretical structure of the scale has been described in the introduction section.

### Data analysis

In the first phase of data analysis, exploratory factor analysis was performed, implementing the FACTOR 9.2 program [46] to determine the dimensional structure of the scale.

In a second phase, the *Rasch Rating Scale Model* (RSM) [47–51] was used and implemented in the WINSTEPS v. 3.73 program [52, 53]. The RSM specifies the probability P_nij_, that a person *n* with ability level β_n_ would be observed in category *j* of a rating scale applied to item *i* at a difficulty level (or ‘endorsability’) δ_*i*_ as opposed to the probability P_ni(j-1)_ of the person’s being observed in category *(j -1)*. Thus, on a Likert scale, *j* could be *strong,* whereas *j-1* might be *moderate*.

1 $$ { \log}_e\left(\raisebox{1ex}{${P}_{nij}$}\!\left/ \!\raisebox{-1ex}{${P}_{ni\left(j-1\right)}$}\right.\right)\kern0.5em =\kern0.5em \beta \kern0.5em -\kern0.5em {\delta}_i\kern0.5em -\kern0.5em {\tau}_j $$

In equation (1), τ_j_ is the *Rasch-Andrich threshold*, also called the *step calibration* or *step difficulty*. The model is appropriate for estimating the latent variable (resilience) and the item endorsability for responses scored in two or more categories. The model also assumes that the distance between the threshold parameters is constant across all items [52, 38].

## Results

### Exploratory factor analysis

To determine the factor structure of the CD-RISC, the *unweighted least squares* (ULS) method was used as the extraction procedure; an oblique rotation (direct oblimin, delta =0) was selected because previous research had shown that resilience factors have moderate-to high-correlations (e.g., Burns and Anstey [32]). The analysis was performed on the matrix of polychoric correlations, reflecting the ordinal nature of the input data. The adequacy of the data was confirmed by Bartlett’s test of sphericity (df =276, p <0.001), the Kaiser-Meyer-Olkin (KMO) index (.94) and the determinant of the matrix (p <0.001).

Initially, item 3 was removed (‘Sometimes fate and God can help’) for having a corrected homogeneity index < .20. To determine the number of factors to be retained, the Kaiser-Guttman rule (eigenvalues > 1.00) was used, resulting in the retention of four factors, which explained 37.2 % of the total variance. However, the fourth factor had an eigenvalue of 1.02, explaining 4.2 % of variance, and it was composed of only two items (2 and 7). Moreover, cross-loadings of < 0.4 on all factors for items 2, 12, 13 and 22 were observed. Because the retention of four factors was not statistically justified in this case, other criteria were used to guide decision-making. First, residual correlations were analyzed by the root mean square residual (RMSR) statistic; the values obtained were .046, .039, .033 and .031 for models with one, two, three and four factors, respectively. Because RMSR indices < .08 are considered indicative of a good-fitting model [54], following the principle of parsimony, this analysis led to the retention of a single factor. Second, the optimized parallel analysis [55] was used as a criterion for retention, comparing the eigenvalues obtained from the analysis with those obtained from 1000 randomly generated polychoric correlation matrices. In our case, randomly generated eigenvalues obtained from the first factor exceeded those obtained from the analysis, which again suggested a one-factor solution to be the most appropriate. Finally, the minimum average partial (MAP) method [56] was used. It was observed that the lowest average value of partial correlations corresponded to a one-factor solution. In light of the above results, it was decided to perform subsequent analyses assuming a one-factor model.

Under conventional factor analysis, the criterion for item retention was that the factor item loading should be at least .40. As shown in Table 1, two items did not meet the criterion: item 9 (‘Things happen for a reason’) and 20 (‘Have to act on a hunch’). Thus, these items were removed, leaving a final version of the scale composed of 22 items with a unidimensional structure. This single factor accounted for 38.6 % of the variance and has been identified in other studies, such as Burns and Anstey [32], Campbell-Sills and Stein [19], Gucciardi et al. [31] and Notario-Pacheco et al. [36]. The decision to remove items 3, 9 and 20 was supported by the results of the Rasch analysis described in the following sections. Table 1 shows item-factor loadings for the one-factor model. Items with factor loadings < .40 are highlighted.

**Table 1:** Items and factor loadings for the of the Connor-Davison Resilience Scale (CD-RISC)

Item		Factor loading
1	Able to adapt to change.	0.573
2	Close and secure relationships.	0.503
3	Sometimes fate and God can help.	---
4	Can deal with whatever comes.	0.619
5	Past success gives confidence for new challenge.	0.583
6	See the humorous side of things.	0.581
7	Coping with stress strengthens.	0.466
8	Tend to bounce back after illness or hardship.	0.644
**9**	**Things happen for a reason.**	**0.355**
10	Best effort no matter what.	0.601
11	You can achieve your goals.	0.692
12	When things look hopeless, I don’t give up.	0.572
13	Know where to turn for help.	0.469
14	Under pressure, focus and think clearly.	0.469
15	Prefer to take the lead in problem solving.	0.618
16	Not easily discouraged by failure.	0.584
17	Think of self as strong person.	0.671
18	Make unpopular or difficult decisions.	0.621
19	Can handle unpleasant feelings.	0.625
**20**	**Have to act on a hunch.**	**0.306**
21	Strong sense of purpose.	0.627
22	In control of your life.	0.604
23	I like challenges.	0.674
24	You work to attain your goals.	0.663
25	Pride in your achievements.	0.629

Items with factor loadings lower than .40 criterion retention are shown in bold. The items are abbreviated examples retrieved from Connor and Davison (2003) http://onlinelibrary.wiley.com/doi/10.1002/da.10113/pdf

### Assessment of the fit of items and persons

Table 2 shows the results of the Rasch analysis (viz., the locations of the items, standard errors, infit and outfit values, point-biserial coefficients and estimated discriminations of the items), once items 3 (‘Sometimes fate and God can help’), 9 (‘Things happen for a reason’) and 20 (‘Have to act on a hunch’), whose infit and outfit mean square (MNSQ) values were > 1.50, were deleted.

**Table 2:** Estimates for items parameters

Item	Measure	Model S.E	INFIT		Outfit		PTBISERL-EX		ESTIM DISCR
			MNSQ	ZEMP	MNSQ	ZEMP	CORR.	EXP.	
1	-0.62	0.06	0.88	-2.0	0.91	-1.4	.56	.55	1.13
2	-0.71	0.06	1.18	2.6	1.16	2.3	.48	.54	0.79
4	0.48	0.06	0.85	-2.4	0.86	-2.3	.62	.59	1.17
5	-0.26	0.06	1.10	1.6	1.16	2.3	.56	.57	0.87
6	-0.01	0.06	0.94	-0.9	0.95	-0.7	.58	.58	1.07
7	0.45	0.06	1.29	4.2	1.29	4.2	.50	.59	0.64
8	-0.66	0.06	0.85	-2.4	0.84	-2.5	.62	.55	1.20
10	-0.16	0.06	1.71	9.1	1.78	9.8	.38	.57	0.16
11	-0.25	0.06	1.14	2.0	1.14	2.1	.59	.57	0.85
12	-0.15	0.06	0.67	-5.7	0.68	-5.5	.67	.57	1.37
13	0.24	0.06	1.12	1.8	1.14	2.1	.57	.59	0.87
14	0.06	0.06	1.22	3.3	1.27	3.9	.49	.58	0.70
15	0.92	0.06	1.30	4.4	1.30	4.4	.51	.60	0.64
16	0.00	0.06	0.89	-1.8	0.87	-2.0	.62	.58	1.15
17	0.57	0.06	0.85	-2.5	0.87	-2.2	.60	.60	1.18
18	0.08	0.06	0.80	-3.3	0.80	-3.4	.66	.58	1.25
19	0.63	0.06	0.97	-0.5	0.96	-0.6	.63	.60	1.04
21	0.73	0.06	0.82	-3.0	0.82	-3.0	.65	.60	1.21
22	0.10	0.06	0.83	-2.7	0.83	-2.8	.62	.58	1.21
23	-0.02	0.06	0.93	-1.1	0.95	-0.7	.59	.58	1.08
24	0.10	0.06	0.84	-2.6	0.84	-2.6	.67	.58	1.20
25	-0.71	0.06	0.85	-2.3	0.85	-2.3	.63	.54	1.19
Items									
ᅟMEAN	0.00	0.06	1.00	-0.3	1.01	-0.1			
ᅟSD	0.48	0.00	0.23	3.3	0.24	3.4			
ᅟSeparation	7.58								
ᅟReliability	0.98								
ᅟRMSE	0.06								
ᅟRS-MC	-1.00								
Persons									
ᅟMEAN	0.75	0.27	1.01	-0.2	1.01	-0.2			
ᅟSD	0.93	0.03	0.57	1.8	0.57	1.8			
ᅟSeparation	2.94								
ᅟReliability	0.90								
ᅟRMSE	0.31								
ᅟRS-MC	0.99								
ᅟCronbach α	0.91								

*INFIT* Inlier-pattern-sensitive fit statistic, *OUTFIT* Outlier-sensitive fit statistic, *MNSQ* Mean Square, *ZEMP t*-standardized fit statistic, *PTBISERL-EX* Point-biserial correlation excluding the current observation from the raw score, *CORR.* Observed correlation, *EXP* Expected correlation, *ESTIM DISCR* Estimate of the local discrimination, *RMSE* Root-mean-square average of the standard errors, *RS-MC* Raw Score to Measure Correlation. *SD* Standard Deviation; at the bottom of the table are the statistical summaries of the items and persons evaluated

Item polarities indicated that all point-biserial correlations were positive and greater than the recommended value of 0.20, falling in a range of 0.38 to 0.67. Therefore, all items met the critical requirement in the Rasch analysis for being aligned in the same direction on the latent variable.

The item separation index was 7.58, demonstrating that the items discriminated between different levels of resilience between subjects. The overall reliability or *item separation reliability* (0.98) indicated that the items formed a well-defined variable and that the reliability of the location of the items on the scale was good; it also provided evidence for the tenability of the local independence assumption. Low reliability would mean that the sample was not large enough to accurately locate the items on the latent variable. With this sample, item difficulties were estimated very accurately.

Estimates of the subjects were reliable. In an attempt to assess the extent to which the test was able to discriminate among levels in the sample to a degree sufficient for our purpose, the separation index was calculated to be 3.28. This value is roughly equivalent to a Kuder-Richardson (KR)-20 or Cronbach’s alpha value of 0.91 and indicated that the CD-RISC, for the sample studied, discriminated among at least three levels (i.e., subjects with low, medium and high resilience). The *person separation reliability* index was appropriate (0.91).

The raw score-to-measure correlation (RS-MC) values are Pearson’s correlations between raw scores and the entire measure, including extreme scores. It is expected that, when the data are complete, they will be near 1.0 for persons and -1.0 for items (which is true in our case: 0.99 and -1.00 were obtained, respectively).

The average fit and standard deviations of the items were suitable (infit =1.00, SD =0.17; outfit =1.01, SD =0.18). The average fit and standard deviations of the persons were also suitable (infit =1.00, SD =0.23; outfit =1.01, SD =0.24). These results suggested that this set of items satisfied, in principle, the requirements needed to identify the construct of resilience.

**Fig. 1:**
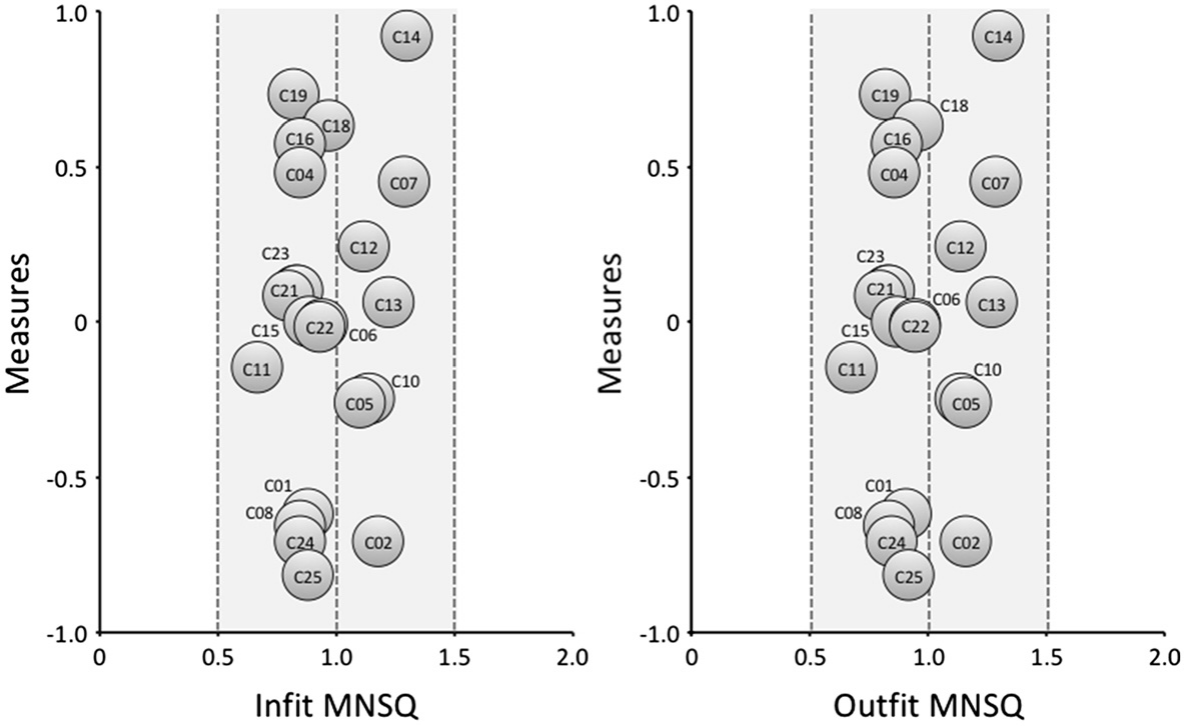
Distributions of items in function of infit mean square (left) and outfit mean square (right) values. The diameter of a bubble represents the magnitude of standard error. The shadow depicts the range of optimal fit. Items 3, 9 and 20 were suppressed

A graphical representation of fit by infit and outfit MNSQ is provided in Fig. 1. The two easiest items (i.e., those most likely to be endorsed) were 25 (‘Pride in your achievements’) and 2 (‘Close and secure relationships’), whereas the most difficult to endorse were numbers 14 (‘Under pressure, focus and think clearly’) and 19 (‘Can handle unpleasant feelings’.) All standard errors were even and reasonably low, as shown by the diameters of the bubbles representing each. Finally, all items were located in the area of 0.5 to 1.5 (areas of acceptable fit), showing their usefulness for measurement [57]. With regard to the subjects, proper model fit was also found: the average infit was 1.01 (SD =0.23), and the average outfit was 1.01 (SD =0.24). It should be noted that only 70 subjects (15.7 %) had values greater than 1.50 for infit or outfit MNSQ. Accordingly, the proportion of persons with good fit amounted to 84.3 % of the sample.

### Specific objectivity

Specific objectivity analysis was carried out by dividing the original sample into two random subsamples of 222 subjects each, followed by the performance of a simple linear regression analysis between the item difficulty parameters obtained from each of them [58]. The correlation between both sets of parameters was .990, with an intercept of 0.001, a slope of 1.115 and a coefficient of determination 0.980. Because the values that would represent perfect fit between the data and the model are 1, 0, 1 and 1, respectively, we concluded that the requirement of invariance of the item parameters was met and that the data showed a good overall fit to the model.

### Appropriateness of the item difficulty level for the sample

**Fig. 2:**
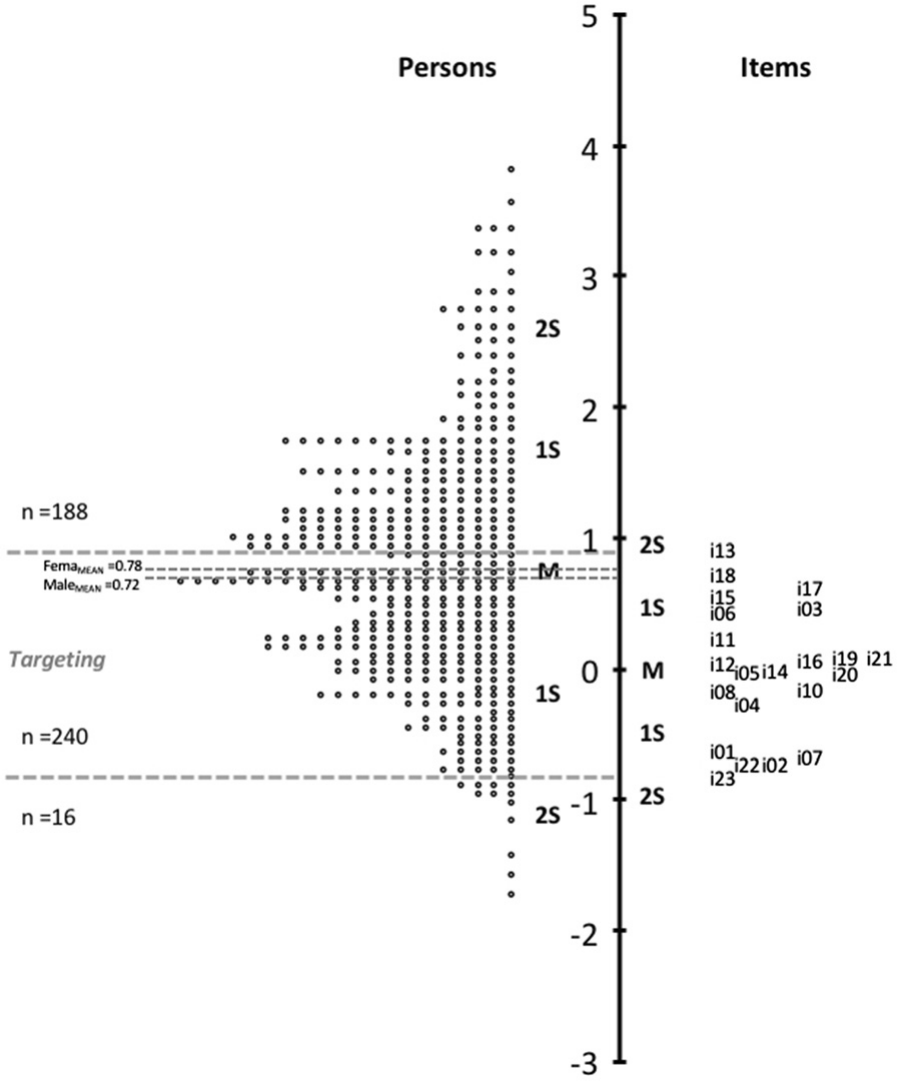
Wright Map (persons and items distributions). M = mean; 1S = standard deviation; 2S = Two standard deviations

The maps of persons and items (Fig. 2), also known as ‘Wright maps’, vividly illustrate how the items on progressively higher difficulty levels overlap with the levels of those persons assessed on the latent trait (resilience). Because the Rasch model uses the same measure (*logit*), both metrics can be compared to determine whether the item difficulty is appropriate for the sample of persons. If the sample is appropriate, there should be considerable overlap in the map between the item difficulty parameters and the levels of the latent trait of persons. This alignment between items and people is called *targeting* in the Rasch analysis jargon.

Figure 2 shows the complete map of items and persons ordered from the highest to the lowest levels. Consequently, persons with high levels of resilience, as well as items most difficult to endorse, are at the top of the map. We can see how the range of the item difficulty parameters partially overlaps the range of the latent trait parameters of persons, indicating that the 22 items assessed subjects with different levels of resilience. However, the following considerations need to be taken into account.

First, the average for persons (*M =0.75, SD =0.93*) was significantly higher than for the items (*M =0.00, SD =0.48*). Second, the amplitude for persons (-1.73 to 3.81 *logits*) was far superior to that for the items (from -0.82 to 0.92 *logits*). Third, a total of 188 subjects (42.7 %) scored above the range of item difficulty, whereas only 16 (3.0 %) did so below that range. The *targeting* area between the item difficulty and the presence of the latent trait in subjects grouped 240 people (49 %). Fourth, no differences were found between the number of men and women in each of the three areas mentioned. An analysis of the standardized Pearson residuals led to the conclusion that the distributions by gender in different areas (upper and lower *targeting* area*s*) were not significantly different from those expected under the equiprobability assumption (*χ*^2^_(2)_ =1.59, p =0.447). This lack of difference was confirmed by an analysis of variance. The means of men and women were 0.72 (*SD* =1.02) and 0.78 (*SD* =0.98), respectively (F_(443)_ =0.531, p =0.467, d =0.025), which denoted a negligible effect size [59]. Moreover, no significant differences were found between the younger subjects and those older than 40 years (F_(443)_ =1.10, p =0.293, d =0.026).

### Dimensionality assessment

One of the underlying assumptions of the Rasch model is that the scale is unidimensional. We checked this requirement using exploratory factor analysis (whose results are detailed in a previous section), infit and outfit statistics, and principal component analysis (PCA) of the Rasch standardized residuals.

When analyzing Rasch models, lack of dimensionality is reflected in poor fit indices. As described above, two fit indices are commonly used: infit MNSQ and outfit MNSQ, both with a theoretical range from 0 to +, which determine to what extent each of the items represents a single underlying dimension. Whereas infit is affected by unexpected response patterns of subjects located near the item position on the scale, outfit is more sensitive to unexpected response patterns of subjects located far from the item location. Because MNSQ is calculated by dividing the value of χ^2^ by the degrees of freedom, *MNSQ* values =1 are ideal, suggesting that the observed variance is equal to the expected variance. Outfit and infit values of 1 + x indicate (100*x)% more variation between observed and predicted patterns by the model from what would be expected if the data and model were to fit perfectly. For example, the infit MNSQ value of 1.12 obtained in item 12 (‘When things look hopeless, I don’t give up’) indicated that this item had 12 % more variation in the observed data than what was predicted by the model. It is assumed [60] that items with MNSQ values > 1.00 have infra-fit (suggesting the presence of unmodeled noise or other sources of variance in the data). An item with a large fit statistic generally indicates that it does not belong to the single construct being measured. In contrast, items with MNSQ values < 1.00 present over-fit (which suggests that there is less variation in the observed data than in the model and, therefore, that the model predicts the data too well, causing inflated summary statistics). As shown in Table 2, infit MNSQ values ranged from 0.88 (‘You can achieve your goals’) to 1.30 (‘Under pressure, focus and think clearly’).

Discrimination indices are shown in the last column of Table 2. When indices are < 1.00, they indicate infra-discrimination, which suggests weak differentiation from one level to the next [60]. In our case, only items 7 and 14 had discrimination indices < 0.70.

In addition to reviewing the MNSQ values described in the preceding paragraphs, we conducted a principal components analysis of the Rasch residuals to determine the unidimensionality of the scale. Principal components analysis decomposes the correlation matrix between items based on standardized residuals (i.e., differences between observed values and those predicted by the Rasch model) to determine whether there are other potential dimensions. The first factor in the analysis corresponds to the Rasch dimension. A variance ≥ 60 % is considered suitable. The second dimension (or first contrast of the residuals) indicates whether there are patterns in the differences of the residuals sufficiently large to suggest the likely existence of more than one dimension. If the variance of the Rasch dimension is low, while being significant in successive contrasts, the scale may be multidimensional. The frequently adopted rule is that the second dimension must include at least 3 items (according to the *eigenvalue*) to be considered as a possible second dimension and should represent at least 5 % of the unexplained variance [60].

**Fig. 3:**
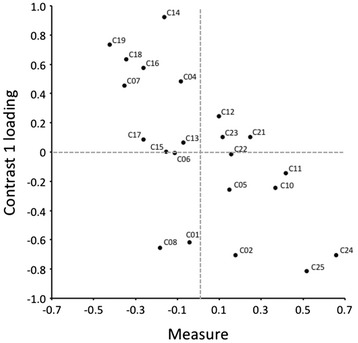
Residual saturation (first contrast)

Principal components analysis of the CD-RISC showed that 39.8 % of the variance was explained by the data. This percentage is almost identical to the variance explained by the model (39.7 %). The first contrast had an *eigenvalue* of 1.9 (lower than the value of 3.0 required to consider a second dimension), which indicated that it contained fewer than 3 items and explained 4.9 % of the variance of the data that was not modeled. Consistent with what has been said, the scale could be considered unidimensional (or ‘sufficiently dimensional,’ in more precise terms), as shown in Fig. 3, in which the existence of distinct clusters to suspect multidimensionality is not evidenced. Figure 3 shows the scatter plot of the measures against loadings in the first contrast to visually illustrate the factorial structure of the CD-RISC. In the chart, the Rasch dimension (in the abscissa) is contrasted with the first contrast factor (on the ordinate). If the items were to appear in separate groups, one might think that the scale did not meet the requirement of unidimensionality.

### Role of the response categories

Each item classification was subsequently reviewed to determine if response categories functioned as expected. First, all frequencies of the four categories used (not true at all, rarely true, sometimes true, often true, true nearly all the time) exceeded the minimum of 10 recommended by Linacre [61]. The most common was *often true* (n =3450) followed by *sometimes true* (n =3346), *true nearly all the time* (n =1767), *rarely true* (n =1354) and *not true at all* (n =295).

The infit MNSQ values were close to the expected value of 1.00 in all categories (1.25, 0.97, 0.96, 0.98 and 0.94, respectively). The outfit MNSQ values were also close to 1.00 in the four categories (1.30, 1.00, 0.98, 0.97 and 0.95, respectively), indicating that the category provided more information (i.e., systematic variance) than noise (i.e., error variance) in the measurement process [61].

**Fig. 4:**
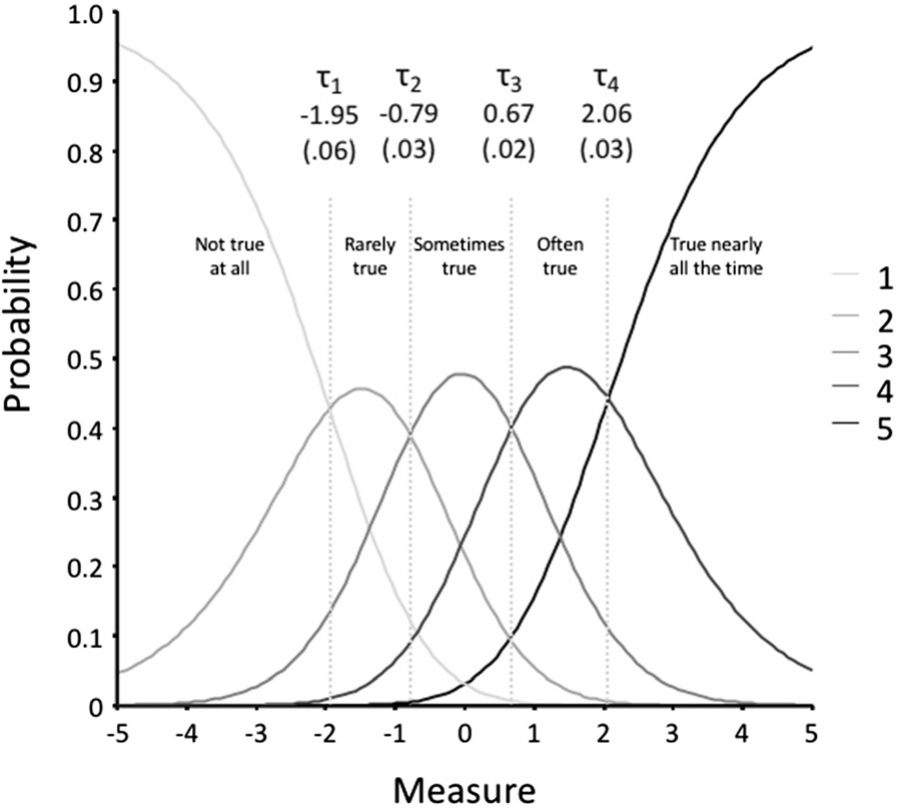
Category probability curves. Vertical lines indicate Rasch-Andrich thresholds (the points at which adjacent categories are equally probable). Parenthesis represents standard error

Secondly, it was confirmed that average measures for all categories advanced monotonically and that there was no particularly noisy category. Thus, the average measures (-0.46, -0.19, 0.38, 1.01 and 1.85) and threshold estimates (-1.95, -0.79, 0.67 and 2.06) showed an increase in parallel with the increase across category labels, suggesting that the categorization of the rating scale was successful (Fig. 4). The sequence was therefore τ_1_ < τ_2_ < τ_3_ < τ_4._ This sequence of values indicated that the Rasch-Andrich threshold parameters were ordered. Therefore, from the *not true at all* category, the most likely transition that occurs is to the *rarely true* category, and so on. Together with the values of these threshold parameters, the standard error of the item steps are shown, showing that the values are relatively low (0.06, 0.03, 0.02 and 0.03).

Looking at the chart of the response category characteristic curves (RCCC), the most likely response category along the continuum can be observed more clearly. This curve relates the probability of item response with its level in the construct measured with the test and is useful in the assessment of item properties. As shown in Fig. 4, the points of intersection between the response categories match the measurement threshold parameters (τ). In turn, these points define regions of most likely responses in the continuum.

### Differential Item Functioning (DIF) analysis

Uniform DIF analysis revealed that there was an item in the scale with a certain DIF risk (in this sample) according to the gender of participants. This was item 2 (‘Close and secure relationships’), and it was 0.71 *logits* more difficult for men (t_(249)_ = -3.43, *p <*0*.*001; MH χ^2^ = 9.44, *p <*0*.*001). DIF size in the other contrasts did not reach 0.50 [52, 62]. With respect to age groups, the only item at risk of DIF was 25 (‘Pride in your achievements’), which was 1.05 *logits* more difficult for older people (*t*_(390)_ = -5.00; *p <*0*.*001; *M-H* χ^2^ = 25.97; *p <*0*.*001).

### Item accuracy

**Fig. 5:**
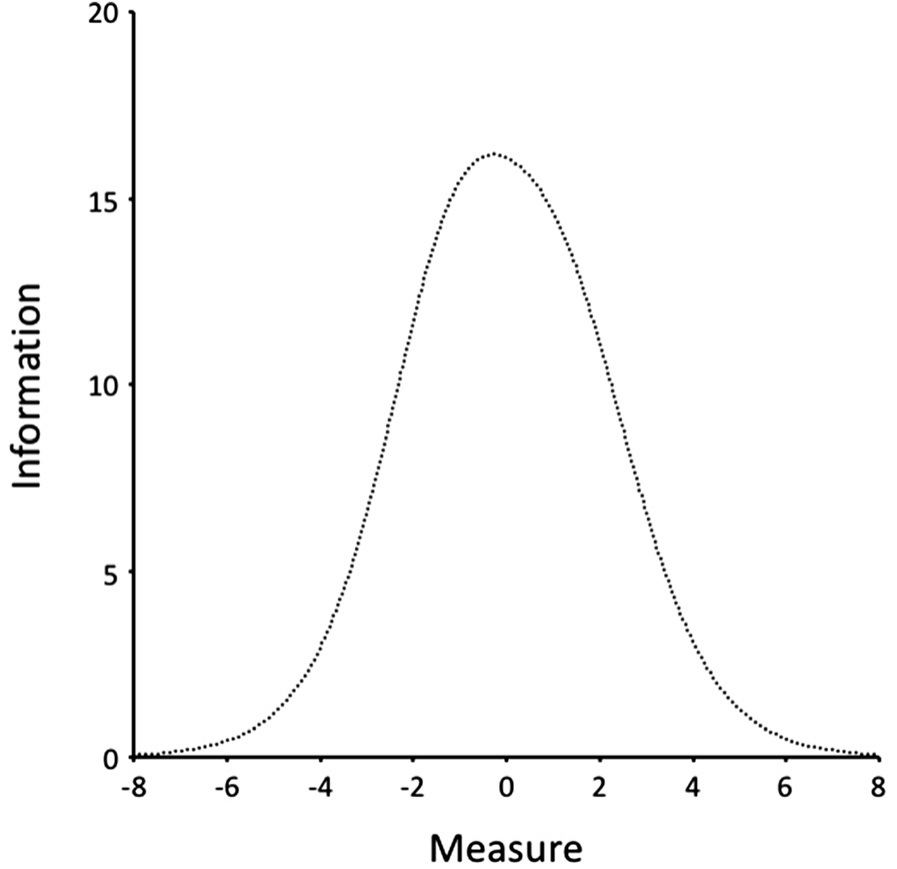
Test Information Function. The curve represents the information provided by complete test (information) at different levels of the latent variable (measure)

With regard to the accuracy of the scores provided by the test items, item information functions and those of the global test were estimated. The result that was obtained was for values of *θ* between *θ* = -1.0 and *θ* =0.5, where test item information scores were highest; hence, it was in this continuum region where the test measured with greater accuracy (Fig. 5). The largest standard errors of measurement occurred at the extremes of the continuum (-6.39 and 6.51).

With regard to the ability of each item to accurately measure different regions of the underlying variable, the results were as follows.

1. Items with maximum information values in the *lower-middle region* of the resilience continuum. In this region were those items that had the highest values of the information function between *θ* = -1.5 and *θ* = -0.5. The items that corresponded with this category were 1, 2, 8, 24 and 25.
2. Items with maximum information values in the *middle*-*middle region* of the resilience continuum. This region was bounded by values of *θ* = -1.0 and *θ* =0.0. Items that had their highest scores in this range were 5, 6, 10, 11, 13, 15, 17, 21, 22 and 23.
3. Items with maximum information values in the *upper-middle region* of the resilience continuum. This region was bounded by the values of *θ* = -0.5 and *θ* =0.5. Items that were included in this region were 4, 7, 12, and 16.
4. Items with maximum information values in the *upper region* of the resilience continuum. Items 14, 18 and 19 were situated in this region, which had maximum information function values between *θ* =0.0 and *θ* =1.0.

### Test rates

The standardized CD-RISC rates were eventually built (qv, Appendix). In them, direct test scores (22 to 110) and measures (i.e., Rasch parameter estimates), along with the standard error for each score, standard scores with its standard error, absolute and cumulative frequencies, and percentiles, are shown. For example, a raw score of 90 on the CD-RISC corresponded to a Rasch measure of 1.35, a standard error of 0.27, a standard score of 595 (*SD* =29) and a percentile of 77.

## Discussion

In this study, we have calibrated the CD-RISC scale using the *Rasch-Andrich Rating Scale Model* (RSM). To our knowledge, the CD-RISC has not been psychometrically calibrated and validated by methods included in item response theory. This methodological framework offers assessment instruments a number of advantages not offered by the traditional analysis methodology (i.e., classical test theory), such as parameter invariance, the estimated accuracy of the items and test, the estimate independence for the test, the joint measurement of items and persons, specific objectivity, range properties and specificity of the standard error of measurement, and the customization ability of the tests. We consider this approach appropriate because psychometric goodness of the original scale was insufficiently substantiated [7] and analysis by Item Response Theory (IRT) models allows considerably deeper understanding of the psychometric properties of the items and scale.

The results showed that the data, taken together, met the requirements of the RSM fit statistics. A good overall fit of persons and items to the model was found: the items of the CD-RISC allow the identification of a relatively wide range of behaviors evaluating resilience. Moreover, both the average reliability indexes of the items and persons and the overall reliability index were found to be acceptable. Moreover, the fit of items was conducted in two phases. In the first, after removing three items with unacceptable infit or outfit values, the fit with the remaining 22 items of the CD-RISC was studied. The results revealed that they all exhibited fit appropriate to the expectations of the model. Therefore, we consider that the data collected with the 22 items could be conveniently explained by the RSM.

The fit for persons showed that, for 84.23 % of individuals (collectively considering mismatches identified by infit and outfit MNSQ), the application of RSM to all items of the CD-RISC could conveniently explain response patterns. Therefore, it can be stated that the scale is useful for measuring resilience in the population for which the scale was administered. In 70 (15.77 %) subjects, the response patterns did not conform to what was expected by the model. Because this was a small percentage, as mentioned above, we retained an interpretation that the model adequately explained the response patterns given by persons to all items. Moreover, the application of RSM to all items allowed the investigation of other item properties, such as measurement error, RCCC and item position on the resilience continuum.

With respect to whether the items were homogeneously and hierarchically ordered with respect to the latent variable evaluated, scale items were shown to be distributed along the continuum, without excessive distance between them, so that, in principle, it would not be necessary to rebuild the instrument for adding items to fill those information gaps. The results thus indicate that the items are distributed in a hierarchical manner and with proper scaling.

The operation of the response categories and their function information were suitable. As illustrated, the RCCC showed that the response categories were ordered on all items, as required by the model [51].

The area alignment of the items that made up the CD-RISC roughly corresponded to more than half of the subjects in the sample. Just over one-third were above the range of item difficulty and only 3 % were below. In other words, the test had a clear ceiling effect with this sample. Given the distribution of item difficulty, the scale seems adequate to measure middle and lower ranges of the latent variable. In this sense, the data support, in part, one of the objectives with which it was built: i.e., assessing resilience in clinical settings and vulnerable persons [14]. The obvious ceiling effect and the absence of items able to adequately discriminate at high levels of the latent variable causes the scale to be inadequate for use in contexts in which the detection of persons with high level of resilience is of interest (for example, in selection processes for certain professions). An interesting theme for future research would be to generate a bank of items suitable for measuring resilient behavior at high levels of the variable.

In relation to the analysis of differential item functioning, we found that, in terms of gender, this study sample was subject to suspected differential operation on one of the items (‘I have stable and close relationships with persons’). Another item also emerged (‘I’m proud of my accomplishments’) as being at risk of DIF in relation to age. In this regard, it should be verified whether DIF in these items was also verified in other samples.

With respect to the accuracy of the scores provided by the test items, information functions of the items and of the overall test were estimated. The consequent result was that, for values of between = -1.0 and =0.5, the highest information function scores of the test were observed; hence, the test measures this continuous region with great accuracy. The largest standard error of measurement was at the extremes of the continuum (-6.39 and 6.51). In future applications of the test and when considering the creation of an item bank, knowing the location of the items on the resilience continuum and where each item provides the maximum information would allow the creation of tests for the desired levels of resilient behavior. In this sense, it would be necessary to prepare items expected to have different endorsability values for the resilience construct for each subcategory of its operational definition. When the aim is to evaluate persons who most likely would have high levels of resilience, a selection of a sample of items should be constructed whose maximum information provided would be in the top positions of the continuum. In contrast, when the Resilience latent variable scores of the persons are low, items could be selected whose maximum information provided would be placed at the bottom of the continuum. Therefore, apart from developing custom tests for accurate diagnosis, having an appropriate item bank would allow one to produce other comprehensive evidence consisting of items that would evenly measure all manifestations of the latent variable.

The following should be noted as limitations of the study. First, the convenience nature of the selection of subjects implies that generalization of the results to the population is not possible. It would be desirable to use probability sampling in future studies to alleviate this limitation. It would also be desirable to use clinical samples to determine if they differ significantly from non-clinical samples. Second, the results showed an apparent CD-RISC ceiling effect, meaning that it is not a reliable test for assessing or detecting high resilience levels. Third, the amount of variance explained by the Rasch dimension was somewhat limited. We believe that this is due to reduced dispersion in item difficulty because the explained variance depended jointly on the dispersion of persons and items. Finally, some overlap should be noted between items in terms of their difficulty (i.e., the difference in *logits* between some of them was very small). However, we think that they should be retained in the scale because their contents refer to clearly distinct concepts while nevertheless relate to the meaning of the latent variable being evaluated.

## Conclusions

The current study shows results of a psychometric analysis of the CD-RISC items’ performance. A good overall fit of persons and items to the model was found: the items of the CD-RISC allow the identification of a relatively wide range of behaviors evaluating resilience, and the average reliability of the scale was aceptable.

It was noted that the CD-RISC presents an essentially unidimensional structure, so that people can be evaluated in a single overall score of resilience. Three of their items showed poor fit to the model, which may mean that they are not associated with the same latent construct as other items.

On the other hand, a clear ceiling effect was observed. Thus, the scale seems adequate to measure middle and lower ranges of the latent variable. This means that this scale, at least in this sample, is not reliable to assess high levels of resilience. In order to improve CD-RISC measurement quality, understanding of resilience and its relationship with other outcomes, it would be necessary to develop a set of items, suitable for measuring resilient behavior at low, middle and high levels of the trait.

## Appendix

**Table 3:** The standardized CD-RISC rates

Score	Measure	S.E.	FREQ.	CUM.FREQ.	CUM.%	PERCENTILE
≤42	≤ -1.81	≥28	0	0	.0	0
≤54	≤ -0.97	≥27	8	8	1.8	1
55	-.91	27	4	12	2.7	2
57	-.78	27	5	18	4.1	3
58	-.72	27	4	22	5.0	5
59	-.65	27	5	27	6.1	6
60	-.59	27	4	31	7.0	7
62	-.47	27	11	42	9.5	9
63	-.41	27	6	48	10.8	10
64	-.34	27	2	50	11.3	11
65	-.28	27	7	57	12.8	12
66	-.22	27	12	69	15.5	14
67	-.16	27	3	72	16.2	16
68	-.10	27	9	81	18.2	17
69	-.03	27	11	92	20.7	19
70	.03	27	11	103	23.2	22
71	.09	27	9	112	25.2	24
72	.15	27	15	127	28.6	27
73	.22	27	15	142	32.0	30
74	.28	27	11	153	34.5	33
75	.34	27	10	163	36.7	36
76	.40	27	9	172	38.7	38
77	.47	27	8	180	40.5	40
78	.53	27	11	191	43.0	42
79	.60	27	13	204	45.9	44
80	.66	28	20	224	50.5	48
81	.73	28	16	240	54.1	52
82	.79	28	6	246	55.4	55
83	.86	28	8	254	57.2	56
84	.93	28	16	270	60.8	59
85	1.00	28	17	287	64.6	63
86	1.06	28	13	300	67.6	66
87	1.13	29	14	314	70.7	69
88	1.21	29	14	328	73.9	72
89	1.28	29	7	335	75.5	75
90	1.35	29	11	346	77.9	77
91	1.42	30	7	353	79.5	79
92	1.50	30	13	366	82.4	81
93	1.58	30	6	372	83.8	83
94	1.65	30	8	380	85.6	85
95	1.74	31	14	394	88.7	87
96	1.82	31	4	398	89.6	89
97	1.90	32	5	403	90.8	90
98	1.99	32	3	406	91.4	91
99	2.08	33	4	410	92.3	92
100	2.18	34	4	414	93.2	93
101	2.28	34	2	416	93.7	93
102	2.38	35	4	420	94.6	94
103	2.49	36	3	423	95.3	95
104	2.61	37	4	427	96.2	96
105	2.73	39	5	432	97.3	97
≥107	≥3.01	≥42	4	436	98.2	98
≥111	≥3.82	≥57	8	444	100.0	99
≥115	≥3.40	≥198	0	444	100.0	100

*SCORE* Direct test scores, *MEASURE* Rasch parameter estimates, *S.E* Standard Error for each score, *FREQ*. Frequency, CUM*.FREQ.* Cumulative frequency, CUM*.%* Cumulative percentage

## Abbreviations

RSM: Rating scale model
ULS: Unweighted least squares
RMSR: Root mean square residual
MAP: Minimum average partial
RS-MC: Raw score-to-measure correlation
PCA: Principal component analysis
MNSQ: Mean square
DIF: Differential item functioning
RCCC: Response category curves
